# Optical System Design for Off-Axis Polarization Super-Resolution Imaging with Four Sub-Apertures

**DOI:** 10.3390/jimaging12070282

**Published:** 2026-06-26

**Authors:** Xiansong Gu, Chao Wang, Huilin Jiang, Boshi Wang

**Affiliations:** 1 National and Local Joint Engineering Research Center for Space Photoelectric Technology, Changchun University of Science and Technology, Changchun 130022, China; emperorgxs@sina.com (X.G.); hljiang_cust@163.com (H.J.); bushwang1205@163.com (B.W.); 2 College of Opto-Electronic Engineering, Changchun University of Science and Technology, Changchun 130022, China

**Keywords:** aberration correction, off-axis optical system, polarization imaging, super-resolution imaging

## Abstract

We propose a dual-aperture, simultaneous-polarization super-resolution imaging system that combines a total internal reflection optical architecture with a digital micromirror device (DMD) for broadband, high-resolution imaging. The system captures multiple polarization states simultaneously with a single detector and offers a compact, lightweight design. Using reflective Wassermann–Wolf differential equations and Seidel aberration theory, we establish astigmatism-correction boundary conditions and apply iterative optimization to jointly correct spherical aberration, coma, astigmatism, and distortion. Because distortion critically affects super-resolution reconstruction by causing mirror–pixel misregistration, we further introduce a custom merit function to tightly constrain chief-ray positions for each sub-aperture and field point on intermediate and final image planes, effectively suppressing distortion. The final design achieves F/2.5, grid distortion below ±0.5%, and near-diffraction-limited performance in all polarization channels. Tolerance analysis of the four sub-apertures confirms that imaging requirements are satisfied, demonstrating robust high-resolution polarization imaging across multiple polarization states.

## 1. Introduction

Polarization detection is an emerging sensing technique that exploits differences in the polarization properties of scattered light, background light, and target light. It provides key advantages, including visibility through clouds and fog, target enhancement, and discrimination between real and false objects [1]. Simultaneous polarization imaging acquires all polarization states in a single exposure, ensuring identical illumination and radiation conditions across channels. This makes it highly suitable for rapidly changing targets and has made it the mainstream approach in polarization detection [2].

To satisfy both simultaneous imaging and miniaturization, polarization imaging systems are mainly divided into focal-plane segmented systems and aperture-divided systems. Focal-plane segmentation is structurally simple but suffers from instantaneous field-of-view mismatch, which is difficult to correct in post-processing. Aperture-divided systems use one focal plane array and projection optics to map multiple polarization-direction images from the same field of view onto different detector regions, enabling simultaneous multi-direction acquisition without field-of-view mismatch. Its main drawback is reduced spatial resolution, which can be compensated by computational super-resolution methods.

In an aperture-divided system design, Pezzaniti and Chenault (2005) developed a mid-wave infrared detector using a relay lens to project four identical images onto one focal plane [3]. Moultrie et al. (2007) developed a low-light polarization imager that simultaneously acquires four sub-images on a single CCD [4]. Leon et al. (2007) designed a 632.8 nm aperture-divided imager that outputs DOP, DOLP, DOCP, and ellipticity from a single exposure [5]. He Hucheng et al. (2014) derived the relationship between system eccentricity and front/rear focal lengths using paraxial imaging theory, and applied the PW method for initial structural design [6,7]. Xujie Huang (2017) designed an MWIR aperture-divided imaging polarimeter (f = 68 mm, F/2, FOV 4° × 3.2°) consisting of a co-aperture Galilean telescope, four sub-aperture double-Gauss objectives, and a co-aperture Cooke triplet relay lens [8]. Wang Qi et al. (2018) designed an infrared aperture-divided polarization imager using combined common-aperture and sub-aperture strategies (3.7–4.8 μm, f = 200 mm, F/4, half-field angle 2°) [9]. Liu Zunbei et al. (2021) proposed an aperture-divided ultraviolet multiband imaging system with a front aperture-division and rear image-combination architecture, covering 240–280 nm, 308 nm, 300–360 nm, and 390 nm [10].

Most of the above designs are transmissive aperture-divided systems. However, in infrared bands with high material absorption, transmissive structures cannot adequately meet wideband high-throughput requirements. Therefore, reflective polarization detection systems are urgently needed. Compared with [9], it is more compact and provides a broader spectral range. In recent years, DMD-based computational imaging and polarization super-resolution have also attracted increasing attention. Xu et al. proposed a polarization super-resolution imaging method based on deep compressed sensing by introducing a DMD into the polarization imaging system [11].Comparison with previous aperture-segmented polarimeters is shown in Table 1.

This paper presents the design principles and methodology of an aperture-divided off-axis simultaneous polarization super-resolution imaging optical system. The system adopts a fully reflective secondary imaging architecture with wide operating bandwidth, no central obscuration, high energy utilization, and good miniaturization potential. It can simultaneously acquire MWIR and LWIR information in four polarization directions, improving target-background discrimination and feature extraction in low-contrast and strong-interference environments (e.g., haze and smoke). The unobscured reflective configuration improves optical efficiency and supports lightweight implementation for airborne and spaceborne applications. The main trade-off is that higher spatial resolution comes at the cost of temporal resolution. Based on Wassermann–Wolf theory and Seidel aberration theory, this work develops initial aberration-correction principles and global optimization methods, and finally presents the design and image-quality evaluation of a large-aperture off-axis fully reflective super-resolution imaging system with a DMD.

Mathematically, the DMD binary coding process can be expressed as

(1) y=Φx+n

where x denotes the high-resolution scene vector, y is the low-resolution measurement vector, Φ is the binary observation matrix generated by the DMD micromirror patterns, and n represents measurement noise. For Bernoulli coding, each element of Φ satisfies Φij∈{0,1}, P(Φij=1)=p, P(Φij=0)=1−p.

To guarantee stable compressed-sensing reconstruction, the observation matrix should satisfy the restricted isometry property (RIP), namely

(2) $$(1-\delta k)\|x{\|}_{2}^{2}⩽\|\Phi x{\|}_{2}^{2}⩽(1+\delta k)\|x{\|}_{2}^{2}$$

where δk is the restricted isometry constant. This condition indicates that the DMD-generated binary observation matrix approximately preserves the energy of sparse signals, thereby bridging the optical coding hardware and the subsequent OMP-based reconstruction algorithm.

## 2. Aperture-Divided Off-Axis Simultaneous Polarization Super-Resolution Imaging System Working Principle

**Figure 1:**
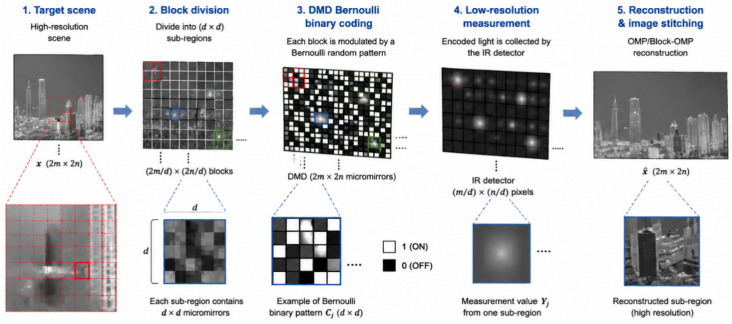
Schematic diagram of DMD-based block compressed-sensing image encoding and reconstruction.

Based on the principle of simultaneous polarization super-resolution imaging and the theory of compressed sensing, a wide-band simultaneous polarization super-resolution imaging system is developed. The key components of the system include: a split-aperture off-axis reflective free-form optical system, a DMD, an infrared polarization focal plane detector, and a computational super-resolution reconstruction unit. A schematic of the system is shown in Figure 1.

Each component in Figure 1 is designed to enable high-resolution polarization imaging: the split-aperture off-axis reflective free-form optical system captures multi-aperture light; the DMD encodes the intermediate image with a block-based compressed sensing pattern; the infrared polarization focal plane detector measures multi-frame low-resolution images; the computational reconstruction unit combines these to achieve d-fold super-resolution, improving spatial resolution and enabling detailed polarization state analysis.

In this work, “super-resolution” refers to the recovery of a higher-resolution image from compressed low-resolution measurements using DMD-based coded sampling and compressed-sensing reconstruction, rather than the development of a dedicated deep-learning super-resolution algorithm.

The aperture-divided off-axis simultaneous polarization super-resolution imaging optical system consists of two main subsystems: a telescope objective system and a relay reflection system. Light from a distant scene passes through the four sub-pupils of the telescope objective system and is imaged onto four equal areas of the intermediate image plane. The DMD is employed to encode the light intensity at the intermediate image plane [12,13]. After encoding, the four beams of light are reflected by the DMD and projected onto the four regions of the infrared polarization focal plane detector, following uniform passage through the relay reflection system.

In each of the four regions of the focal plane, polarization is analyzed by attaching a broadband polarization grating oriented in different directions: I_(0)_, I_(45)_, I_(90)_, and I_(135)_. The DMD encoding is performed sequentially for each polarization state, generating multi-frame low-resolution intensity images. These images are then processed through sub-pixel reconstruction, and the image processor outputs super-resolution polarization images for each polarization direction. Finally, the Stokes parameters of the target are computed from the reconstructed super-resolution images [14,15,16].

The Stokes vector $S={\left[I,Q,U,V\right]}^{T}$ is a set of four parameters that fully describe the polarization state. Where I represents the total light intensity, Q represents the intensity difference between 0° and 90° polarization, U represents the intensity difference between 45° and 135° polarization, and V represents the intensity difference between right-handed circular polarization and left-handed circular polarization. In common DoFP (Division of Focal Plane) or polarizer imaging systems, only the intensity in the four linear polarization directions of 0°, 45°, 90°, and 135° can be measured, so I, Q, U can be obtained, but V cannot be directly obtained. Therefore, by constructing Stokes components through intensity difference and sum, the Stokes vector representation of the polarization state can ultimately be obtained.

(3) $$S=\left[\begin{array}{l} S_{0}\\ S_{1}\\ S_{2}\\ S_{3} \end{array}\right]=\left[\begin{array}{l} I_{0}+I_{90}\\ I_{0}-I_{90}\\ I_{45}-I_{135}\\ 0 \end{array}\right]$$

where S0 denotes the total intensity; S1 represents the intensity difference between 0° and 90°; S2 represents the intensity difference between 45° and 135° and if S3 only measures linear polarization, take 0. Based on the reconstructed Stokes parameters, the degree of linear polarization (DoLP) and the angle of polarization (AoP) can be calculated as

(4) $$DoLP=sqrt\left({S1}^{2}+{S2}^{2}\right)/S0$$

(5) AoLP=0.5arctan(S2/S1)

In order to evaluate the polarimetric performance of the system, the polarimetric accuracy can be defined as the deviation between the reconstructed and reference DoLP values, namely ΔDoLP = |DoLP_rec − DoLP_ref|. The angular error can be defined as the deviation between the reconstructed and reference AoP values, namely ΔAoP = |AoP_rec − AoP_ref|. The polarimetric sensitivity can be characterized by the minimum detectable change in DoLP or AoP under a given signal-to-noise ratio. These metrics provide the basis for subsequent experimental calibration and polarimetric performance evaluation. Since the present work focuses on optical design and simulation-based feasibility evaluation, quantitative measurement of these polarimetric metrics using calibrated experimental data will be carried out in future prototype tests.

The encoding principle of this system is centered on the compressed sensing theory, and it achieves super-resolution effects by relying on the sparsity characteristic of signals. Considering the super-resolution requirement of this study for area-array detectors, a “block-based” compressed sensing strategy is specifically adopted. The specific process is as follows: first, the target scene is divided into multiple sub-regions and projected onto the DMD. Each sub-region consists of d × d micromirrors, and the DMD completes the encoding and sampling of these sub-regions simultaneously. Next, the low-resolution area-array detector captures the encoded image information, with each pixel of the detector corresponding to one sub-region in a one-to-one manner. Subsequently, a reconstruction algorithm is used to process the collected image data, realizing high-resolution restoration of each sub-region. Finally, all the reconstructed sub-region images are stitched together to form a complete target scene image, achieving d-fold super-resolution image reconstruction.

In this work, the sparsity assumption refers to the fact that the target scene can be represented as a sparse vector in a transform domain Ψ (e.g., wavelet or DCT basis), where only k ≪ N coefficients are non-zero. The system further assumes block-wise sparsity, meaning that each d × d sub-region is sparse independently. Accurate reconstruction requires that the sparsity level k satisfies k < m/log(N), ensuring stable recovery under the OMP framework. The low-resolution detector refers to an infrared focal plane array (FPA). Its effective spatial resolution is intentionally reduced through block-wise optical mapping in the relay imaging system, such that each detector pixel integrates the light intensity from a d × d micromirror sub-region on the DMD.

In the DMD encoding process of this system, the Bernoulli encoding strategy is one of the core implementation methods. Based on the Bernoulli random distribution, this strategy assigns several sets of binary encoding states to the d × d micromirror units in each sub-region on the DMD. The probability that a micromirror is in the “on” state is set to p (usually 0.5 to ensure the randomness and uniformity of encoding), and the probability of being in the “off” state is 1-p. Its random binary encoding characteristic can ensure that the observation matrix Φ satisfies the “Restricted Isometry Property (RIP)” required by compressed sensing, effectively reducing the correlation between encoded signals, thereby guaranteeing the quality of image reconstruction.

For the selection of the reconstruction algorithm, the system adopts the Orthogonal Matching Pursuit (OMP) algorithm [17]. In essence, this algorithm solves the optimization problem of l1-norm minimization. By approximating the l1-norm to the l0-norm and combining it with the improved Block Orthogonal Matching Pursuit (Block OMP) algorithm, the reconstruction accuracy is improved. The specific process is as follows: first, the DMD with 2 m × 2 n pixels is divided into (2 m/d) × (2 n/d) blocks. The same coding pattern Cj is applied to each block, and the light intensity Yj is captured. A set of observation vectors Y is obtained through matrix transformation. Then, with the observation vector Y, the observation matrix Φ (sensing matrix), the sparse transformation matrix Ψ, and the signal sparsity level k as inputs, the OMP algorithm is initiated. The key index is determined by calculating the inner product between the residual and the columns of the observation matrix; the index set is updated, and the least squares solution is obtained. The residual is updated iteratively until the termination condition t > k is met. Finally, the reconstructed sub-block images are combined to form the final image with a resolution of 2 m × 2 n.

In practical experiments, factors such as coating quality, surface figure accuracy, and assembly precision have a pronounced impact on the polarization measurement accuracy of the optical system. Therefore, we typically employ polarization calibration to comprehensively mitigate the effects of distortion, coating, surface figure, and assembly on the Stokes parameters.

Since the proposed system is an off-axis reflective optical system incorporating a DMD, polarization aberrations should also be considered in addition to conventional scalar image-quality metrics. Multiple oblique reflections, coating-induced phase retardance, unequal reflectance for s- and p-polarized components, and the micromirror tilt of the DMD may introduce diattenuation, retardance, and Stokes-parameter crosstalk [18]. These effects may further influence the accuracy of DoLP and AoP reconstruction, especially in a broadband MWIR–LWIR system. Therefore, MTF, spot size, and grid distortion can only verify the geometric and radiometric imaging feasibility of the optical system, but they cannot fully characterize the final polarimetric accuracy. In future prototype development, a vector polarization ray-tracing model based on Jones/Mueller calculus will be established to evaluate the polarization aberrations introduced by the reflective mirrors and the DMD. The calibration method reported in Ref. [14] will also be used as an important reference for suppressing polarization aberration and correcting Stokes-parameter errors.

## 3. Principles and Methods of Aberration Correction for the Aperture-Divided Off-Axis Simultaneous Polarization Super-Resolution Imaging Optical System

### 3.1. Aberration Elimination Principle for Optical System Based on Wassermann–Wolf Theory and Seidel Coefficients

The optical system is a fully reflective secondary imaging system. The telescope objective forms the primary image on the DMD, and the relay reflection system re-images this intermediate plane onto the infrared polarization focal plane detector. The main aberration-correction challenge lies in the relay section, which must satisfy two requirements: (i) spherical aberration, coma, astigmatism, and other wavefront errors must be corrected to near-diffraction-limited quality; and (ii) distortion must be suppressed so that an n × n super-resolution coding block can be mapped onto a single detector pixel without chief-ray misregistration. In particular, residual distortion directly affects the pixel-to-micromirror mapping required by compressed sensing reconstruction. If not properly controlled, this misregistration may introduce reconstruction artifacts such as edge blurring and polarization crosstalk. Recent studies have also explored automatic generation of starting points for freeform imaging optical systems. For example, Chen et al. proposed a deep-learning-based framework for generating starting points for highly nonrotationally symmetric freeform refractive, reflective, and catadioptric systems [18]. Different from such data-driven approaches, the present work derives the initial relay-reflection structure from reflective W-W differential equations and Seidel aberration theory, which provides a physically constrained starting point for subsequent optimization.

Due to the lack of readily available proprietary data as a starting point for the design of relay-reflection optical systems, the classical Wassermann–Wolf (W-W) design theory [16] was further developed and applied to the design of the optical initial structure of the system. Derive the W-W equations for the relay reflection system, and solve for a pair of W-W surfaces with spherical aberration, coma, and astigmatism.

**Figure 2:**
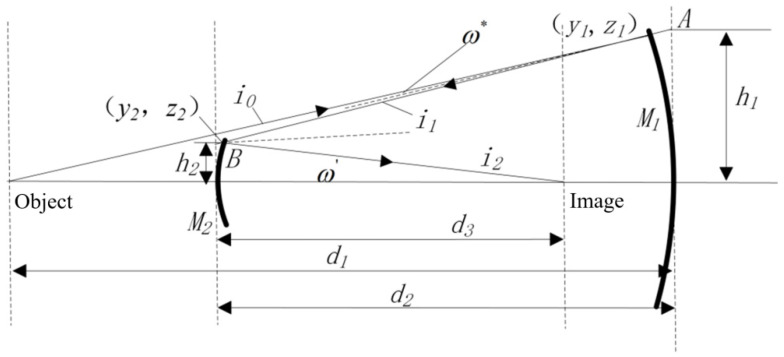
W-W model of coaxial two-mirror system.

A coaxial reflective W–W model consisting of mirrors M_2_ and M_3_ is shown in Figure 2. Light enters from the left, reflects from M_2_ and M_3_, and reaches the image plane. Here, i_0_ is the incident ray on M_2_, i_1_ is the incident ray on M_3_, and i_2_ is the emergent ray after reflection from M_3_. The ray coordinates in the meridional plane are written in parametric form as y = y(t) and z = z(t). Let (y_1_, z_1_) and (y_2_, z_2_) denote the intersection points of i_0_ on M_2_ and i_1_ on M_3_, respectively. The aperture angles of the chief ray at M_2_ and M_3_ are denoted by u_1_ and u_2_. The longitudinal coordinates of the intersections of the chief-ray extensions with the vertex tangents of M_2_ and M_3_ are denoted by h_1_ and h_2_. The axial separations are defined as follows: d_1_ is the distance from the object plane to M_2_, d_2_ is the distance from M_2_ to M_3_, and d_3_ is the distance from M_3_ to the image plane.

Combining the sinusoidal condition with the law of reflection, a set of coaxial two-reflector W-W differential equations is derived.

(6) $$\frac{dz_{1}}{dt}={\left(\frac{R\cos\omega *+R_{z}}{R\sin\omega *+R_{y}}+\tan\omega *\right)}^{-1}\times \left(\frac{dh_{1}}{dt}-z_{1}\frac{d\left(\tan\omega *\right)}{dt}\right)$$

(7) $$\frac{dz_{2}}{dt}={\left(\frac{R\cos\omega^{\prime }+R_{z}}{R\sin\omega^{\prime }+R_{y}}+\tan\omega^{\prime }\right)}^{-1}\times \left(\frac{dh_{2}}{dt}-z_{2}\frac{d\left(\tan\omega^{\prime }\right)}{dt}\right)$$

where $R_{z}=d_{2}+z_{2}-z_{1}$, $R_{y}=y_{2}-y_{1}$, $R=\sqrt{R_{y}^{2}+R_{z}^{2}}$, $y_{1}=h_{1}-z_{1}\tan\omega *$, $y_{2}=h_{2}-z_{2}\tan\omega^{\prime }$.

In solving Equations (1) and (2), the sinusoidal condition must also be satisfied.

(8) $$\frac{\sin\omega *}{\sin\omega^{\prime }}=C$$

where ω* is the aperture angle of the incident ray, $\omega^{\prime }$ is the aperture angle of the outgoing ray, *C* is a constant.

In the W-W two-reflector design process, initial values for *d*_1_, *d*_2_, and *d*_3_ are selected based on the system’s dimensional constraints to ensure a compact configuration with suitable optical reach. The parameter *d*_1_ primarily governs the overall length of the relay reflection system, while *d*_2_ and *d*_3_ are initially assigned values equal to *d*_1_ to serve as a baseline for further optimization.

The process begins by tracing the chief ray from the object space to the tangent plane of M_1_, recording the values of ω* and *h*_1_. Similarly, in the image space, the chief ray is traced backward from the image point to the tangent plane of M_2_, storing the corresponding $\omega^{\prime }$ and *h*_2_. Next, the derivatives $\frac{d\tan\omega *}{dt}$, $\frac{d\tan\omega^{\prime }}{dt}$, $\frac{dh_{1}}{dt}$ and $\frac{dh_{2}}{dt}$ are computed.

The initial conditions are set as z_1_ = 0, y_1_ = 0, and z_2_ = d_2_. The fourth-order Runge–Kutta method is then used to solve Equations (2) and (3) iteratively for z_1_, z_2_, y_1_, and y_2_. This process is repeated until all sampled rays are traced. The resulting discrete points are fitted to an even-order aspheric surface model. The surface sag of M_2_ and M_3_ is expressed as

(9) $$s_{1}=\frac{\gamma_{1}^{2}}{r_{1}+\sqrt{r_{1}^{2}-\left(1+e_{1}\right)\gamma_{1}^{2}}}+\sum a_{i}\gamma_{1}^{2i}$$

(10) $$s_{2}=\frac{\gamma_{2}^{2}}{r_{2}+\sqrt{r_{2}^{2}-\left(1+e_{2}\right)\gamma_{2}^{2}}}+\sum a_{i}\gamma_{2}^{2i}$$

where r_1_, r_2_ are the radius of curvature of mirror M_1_ and mirror M_2_ respectively; e_1_, e_2_ are the quadratic coefficients of the surfaces of mirrors M_1_ and M_2_ respectively; γ is the radial coordinate of the perpendicular optical axis; and $a_{i}\gamma^{2i}$ is the higher term of the aspherical surface.

Next, the distortion elimination equation is derived. According to Seidel’s aberration theory, the PW form of the primary aberration is given by

(11) $$S_{5}=\sum \frac{y^{2}}{h}P-2J\sum \frac{y}{h}W+J^{2}\sum \phi +\sum h^{2}y^{2}K$$

where *y* is the height of the main ray on the mirror. *J* is the Abbe constant of the optical system; *h* is the height of the fringe field of view ray on the mirror. ϕ is the focal power of the optical element. The expressions for *P*, *W*, and *K* are respectively:

(12) $$P={\left(\frac{\Delta u}{\Delta 1/n}\right)}^{2}\Delta \frac{u}{n}$$

(13) $$W=\left(\frac{\Delta u}{\Delta 1/n}\right)\Delta \frac{u}{n}$$

(14) $$K=-\frac{e^{2}}{r^{3}}\Delta n$$

where ∆*n* is the difference between the refractive indices of the object-side and image-side media. ∆*u* is the difference between the object-side and image-side aperture angles of the optical system. *r* is the radius of curvature of the mirror. *e* is the quadratic coefficient of the mirror surface.

Let *ɑ* denote the obstruction ratio of mirror M_2_ to M_1_, *β_2_* denote be the magnification of mirror M_2_, and *β* denote be the magnification of the whole relay reflection system. The radius of curvature of the mirror and the *P*, *W*, and *K* parameters of the PW method are expressed by *ɑ* and *β*_2_, and are substituted into Equation (6) to obtain:

(15) $$\begin{array}{l} S_{5}=-\frac{\beta_{2}^{3}}{4}+\frac{{\left(1-\alpha \right)}^{2}{\left(1-\beta_{2}\right)}^{3}}{4\beta_{2}^{4}}-\frac{3\beta_{2}^{2}}{2}-\frac{3{\left(1-\alpha \right)}^{2}\left(1-\beta_{2}^{2}\right)}{2\beta_{2}^{4}}-2\beta_{2}\\ \;\;+\frac{3\beta_{2}\left(\alpha -1\right)\left(\beta_{2}+1\right)-\alpha \left(\alpha -1\right)\left(\beta_{2}-1\right)}{\alpha \beta_{2}^{3}}\\ \;\;+\frac{e_{1}^{2}{\left(\alpha \beta_{2}+\alpha^{2}\beta -\alpha \beta \right)}^{3}}{4{\left(\alpha^{2}\beta -\alpha \beta \right)}^{3}}-\frac{e_{2}^{2}{\left(1+\alpha \right)}^{3}{\left(\beta_{2}-1\right)}^{3}}{4\beta_{2}\beta^{3}} \end{array}$$

where $\alpha =h_{2}/h_{1}$. *h*_1_, *h*_2_ are the heights of the fringe field-of-view rays on the two mirrors, respectively. *β* = *l*_2_/*l*_1_, where *l*_1_, *l*_2_ are the side dimensions of the target surfaces of the DMD and the infrared polarization focal plane detector, respectively. The magnification *β* is determined by the size of the reflecting surface of the DMD and the target surface of the detector, and is a known quantity.

**Figure 3:**
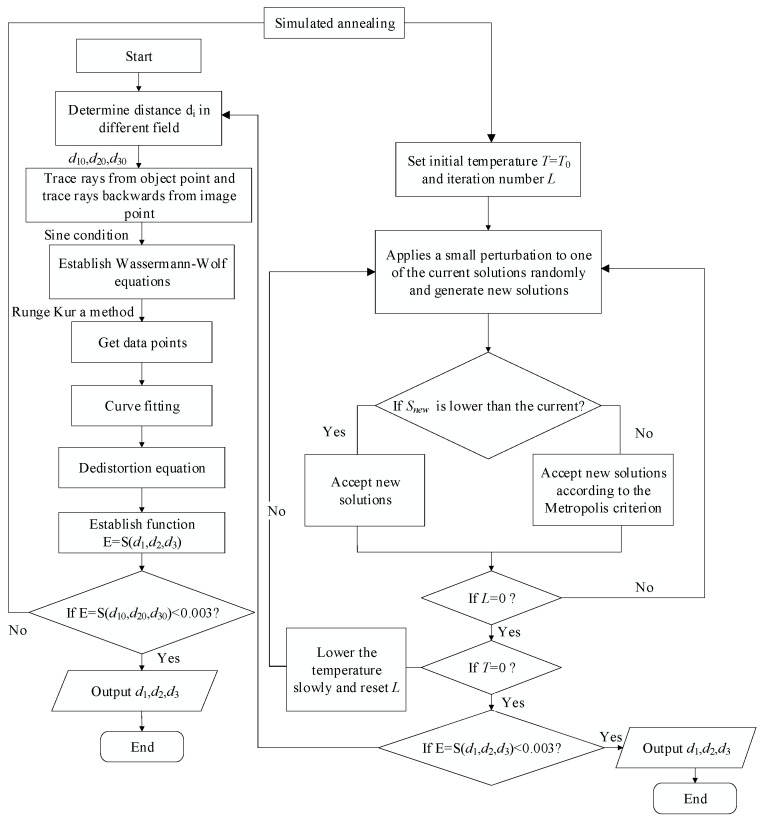
Initial structure design flow chart of relay reflection optical system.

The complete solution flow for the initial structure of the relay reflection system is shown in Figure 3. The *e*_1_, *r*_1_, *e*_2_, *r*_2_ and *d_i_* (*i* = 1, 2, 3) of the two aspherical surfaces after solving and fitting the first W-W equations in the previous section are substituted into Equation (10). The simulated annealing-based multivariate function optimization [19] is used to find a more reasonable value of the system parameter if a larger value of *S*_5_ is obtained. Multiple loops are performed, and in each loop, a perturbation is randomly added to one of the parameters *d*_1_, *d*_2_, *d*_3_, while the other parameters are kept constant. All the other parameters are kept constant. *d_i_* fluctuates within a certain range, $d_{1},d_{2}\in [100,150],d_{3}\in [50,100],d_{2}<d_{1}$. Based on the new *d*_i_, the W-W equation is re-solved to obtain a new *S*_5_, which is accepted if *S*_5_ becomes smaller, or not, according to the Metropolis principle. This cycle continues until the temperature drops to the set termination temperature. At this point the design of the initial structure of the relay reflector system for spherical aberration, coma, dispersion and distortion elimination is considered to be completed.

### 3.2. Design Methods of Aberration Correction for the Aperture-Divided Off-Axis Simultaneous Polarization Super-Resolution Imaging Optical System

#### 3.2.1. Telescope Objective Optical Design

The single-aperture coaxial rotationally symmetric optical path is used as a starting point for designing the overall structure of the telescope objective by increasing the off-axis and tilt amount in the meridian direction of the monolithic reflector, and adjusting the primary image plane to a suitable position. Next, the mirror surface shape is complicated, and the degrees of freedom of the mathematical model of the surface shape are gradually increased (even-order aspherical surface → X-Y polynomial free-form surface), until the image quality of the single-aperture telescope objective approaches the diffraction limit. The telescope objective was designed to split the aperture, and the pupil of the telescope objective was centered in the X and Y directions four times to obtain an optical path with four sub-apertures. In order to minimize the overall size, each aperture needs to be close to each other. Finally, the initial optical structure of the split-aperture telescope objective is obtained.

Due to the need for simultaneous polarization imaging, the four sub-apertures of the telescope objective are required to image four regions of equal size to the DMD at the primary image plane. The four images are arranged in close proximity, with no gaps between the images, to maximize the use of the DMD mirrors. Therefore, in the optimization, it is necessary to control the landing point of the main ray at the DMD for each sub-aperture system.

**Figure 4:**
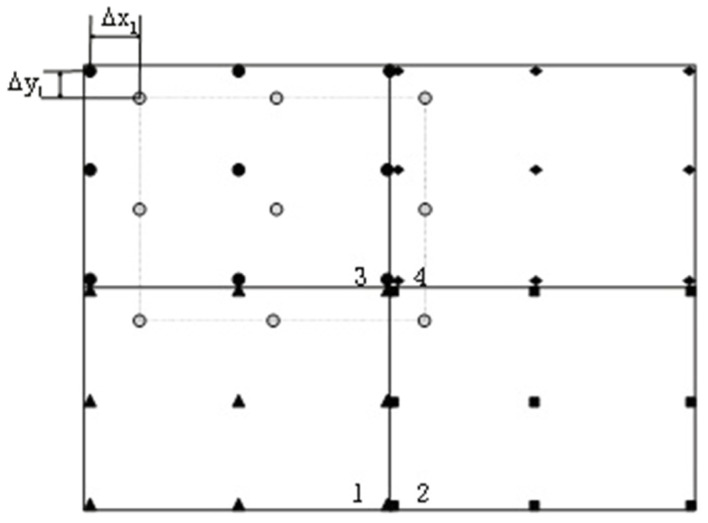
Ideal light spot at the image plane. 1: sub-aperture 1. 2: sub-aperture 2. 3: sub-aperture 3. 4: sub-aperture 4.

As shown in Figure 4, 3 × 3 typical field-of-view sampling points are taken in the image area of each sub-aperture. The black points are the ideal locations of the main light fall-off in each field-of-view, and the gray points are the actual light fall-off points. Since the system is symmetric about the *yoz* plane, only 5 × 3 field-of-view sampling points of sub-aperture 1 and sub-aperture 3 need to be extracted for the calculation.

Use ZEMAX macro language to write a custom optimization evaluation function, use RAGX and RAGY operation numbers to trace the coordinates of the falling point of the ray on the DMD, and obtain the root-mean-square (RMS) distance difference between the falling point of the actual ray and the ideal falling point, and write a custom evaluation function, whose expression is as follows:

(16) $$MF=\sqrt{\frac{\sum r_{i}^{2}+\sum W_{i}\left[{\left(x_{i}-x_{oi}\right)}^{2}+{\left(y_{i}-y_{oi}\right)}^{2}\right]}{1+\sum W_{i}}}$$

where *r_i_* is the radius of the diffuse spot in different fields of view. *W_i_* is the optimization weight value for different field of view sampling points. *x_i_* and *y_i_* are the coordinates of the actual fall point of the main ray for each field of view. *x_oi_* and *y_oi_* are the ideal coordinates of the sampling points for different fields of view, where *i* represents the labeling of the field of view and takes values in the range of 1–15. In the optimization, the values of the modulation transfer function (MTF) of the meridian and arc-vector surfaces and the plumbline aberrations are added to the customized evaluation function. The design of the telescope is completed by optimizing the evaluation function until it reaches a very small value.

#### 3.2.2. Relay Reflector System and Overall Optical System Design

Based on the principle of coaxial system aberration in Section 3.1, the W-W differential equation of the coaxial relay reflection system is listed and solved to obtain a pair of coaxial aberration reflecting surfaces. In order to avoid center blocking and obtain good image quality, the initial structure of the relay reflection system is off-axis. The reflector surface shape is appropriately complicated, and aberrations are controlled using operators such as DISG (DiskGenius3.50), DIMX (dimensionX 3.30), etc., until the image quality of the relay reflector system is close to the diffraction limit.

Finally, the telescope objective is connected to the relay reflection system, and the image plane of the former is used as the object plane of the latter. The surface shape of the primary image plane is changed from a flat surface to a DMD micromirror array surface shape. The telescope objective parameters are kept unchanged, and only the surface shape parameters of the relay reflection system and the eccentricity and tilt of the two surfaces are released as optimization variables. The next step is to write an optimized evaluation function according to Equation (11), so as to make the light fallout of each sub-aperture on the final image plane as close as possible to the ideal value, and to control the overall image quality of the optical system, thus completing the design of the optical system as a whole.

## 4. Design of Aberration Correction for the Aperture-Divided Off-Axis Simultaneous Polarization Super-Resolution Imaging Optical System

### 4.1. Design Results

Based on the design principles and methods described in the previous section, a split-aperture off-axis super-resolution imaging optical system was designed. The main technical specifications are listed in Table 2.

**Figure 5:**
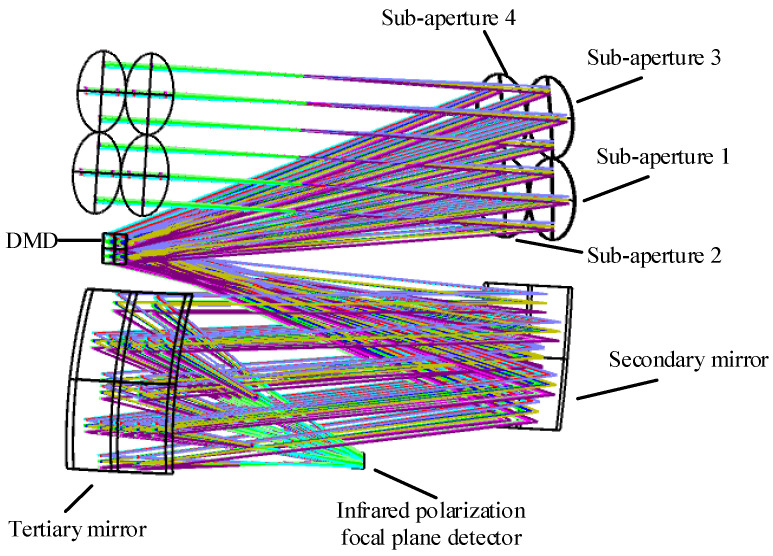
Layout of aperture-divided off-axis simultaneous polarization super-resolution imaging optical system.

The detector used in this imaging optical system is a polarization infrared detector based on the GWIR 0202X1A uncooled mid-wave and long-wave infrared detector of Northern Wide Micro. In the infrared focal plane attached to the different directions of the metal grating polarization, the target surface polarization direction distribution is shown in Figure 1. The three-dimensional optical path of the split-aperture off-axis simultaneously polarized super-resolution imaging optical system is shown in Figure 5. The system focal length is 100 mm. It is compact with a transverse length of 150 mm and a longitudinal length of 147.33 mm.

The optical system consists of four sub-aperture primary mirrors and secondary (M_2_) and tertiary mirrors (M_3_) characterized by X-Y polynomial surface profiles. The M_2_ and M_3_ together form a relay reflection system. Initially, the telescope objective was designed as detailed in Section 3.2.1. Following this, the W-W differential equation was solved using the methodology described in Section 3.1 to obtain discrete surface data points for the secondary and M_3_ (refer to Table 2, where N denotes the number of facet data points).Profile data points of M1 and M2 are shown in Table 3.

**Figure 6:**
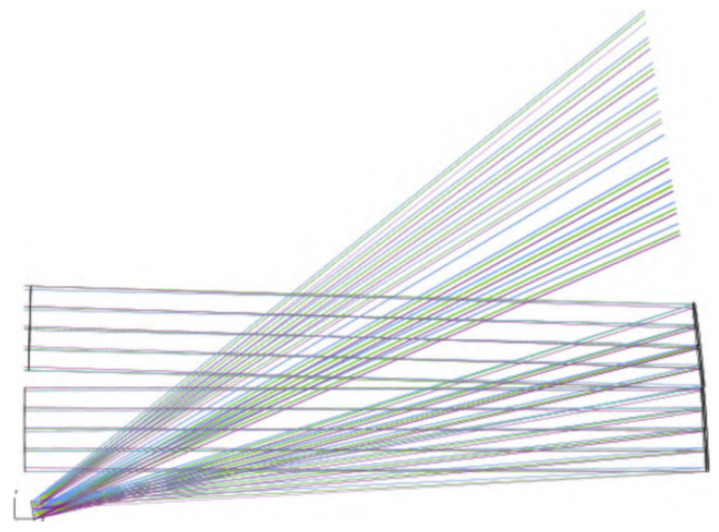
Direction of the light when the DMD micro-mirror is off.

At this stage, the system parameters were set as follows: d_1_ = 150 mm, d_2_ = −144.122 mm, and d_3_ = 94.67 mm. The mirror surface data were processed using our custom surface-fitting program based on the approach outlined in Section 3.1. The resulting W-W surface pair was then imported into the ZEMAX (Zemax2021) optical design software to establish the initial configuration of the relay reflection system. Finally, the M_2_ and M_3_ were optimized in an off-axis layout to eliminate any obstruction in the optical path. The telescopic objective is connected to the relay reflection system, and a DMD surface is introduced at the primary image plane, which has several micromirrors with both on and off states. In the on state, the reflected light from the DMD enters the relay reflection system normally. In the off state, all the light rays from the DMD are reflected to the inner wall of the mirror tube, as shown in Figure 6. In this case, the inner wall of the mirror can be blackened to absorb light in the abnormal optical path and prevent stray light from entering the detector. The field of view of each sub-aperture was expanded to cover the entire DMD area, and the final result was optimized by no more than 10 iterations in the optical design software. The parameters of each mirror are shown in Table 4.

**Figure 7:**
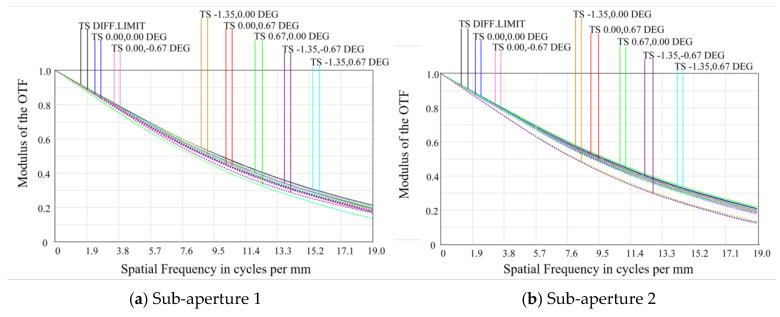

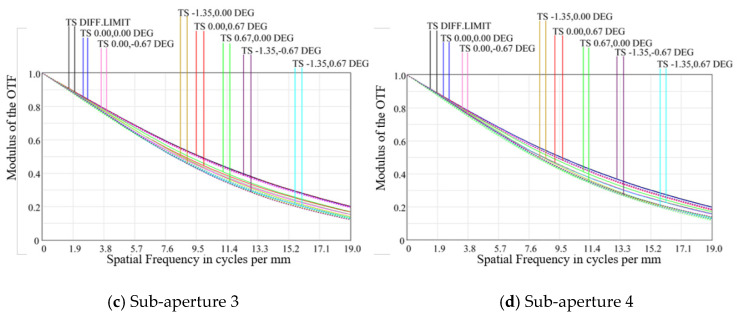
MTF of telescope objective. (**a**) Sub-aperture 1; (**b**) Sub-aperture 2; (**c**) Sub-aperture 3; (**d**) Sub-aperture 4.

**Figure 8:**
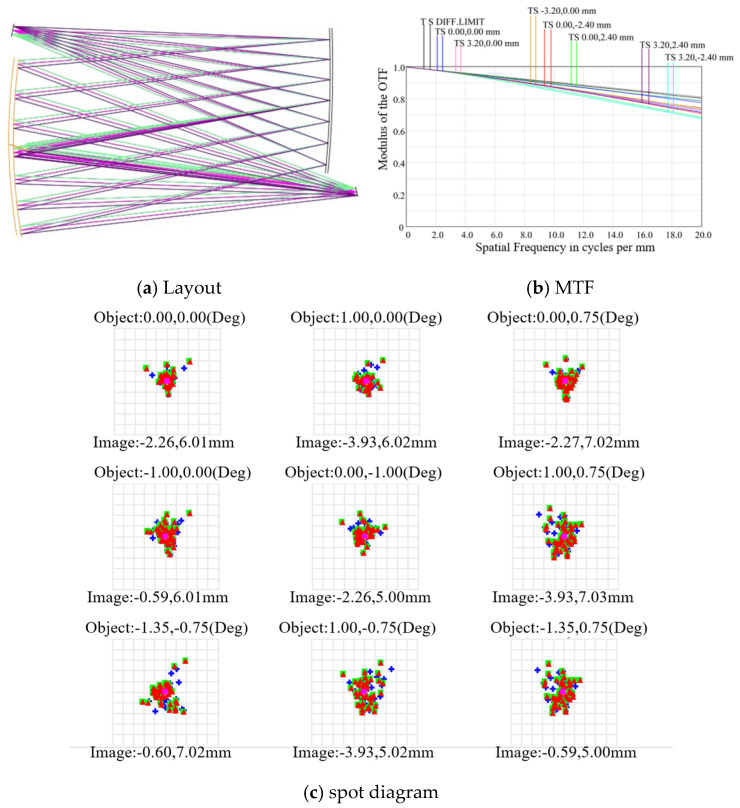
Image quality evaluation of relay reflection optical system. (**a**) Layout. (**b**) MTF. (**c**) spot diagram.

**Figure 9:**
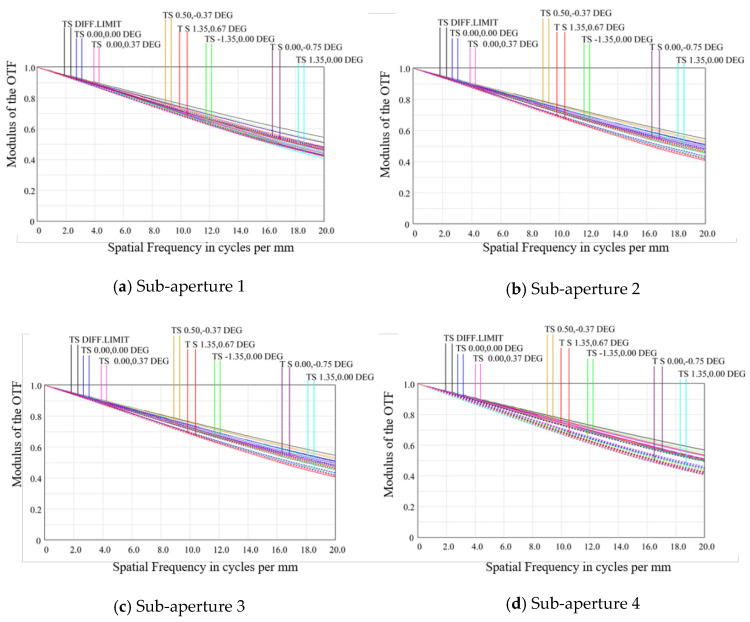
MTF of aperture-divided off-axis simultaneous polarization super-resolution imaging optical system. (**a**) Sub-aperture 1. (**b**) Sub-aperture 2. (**c**) Sub-aperture 3. (**d**) Sub-aperture 4.

**Figure 10:**
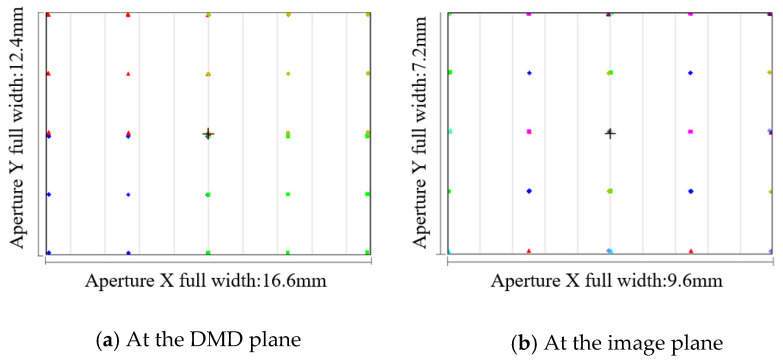
Footprint diagram of aperture-divided off-axis simultaneous polarization super-resolution imaging optical system. (**a**) At the DMD plane.(**b**) At the image plane.

**Figure 11:**
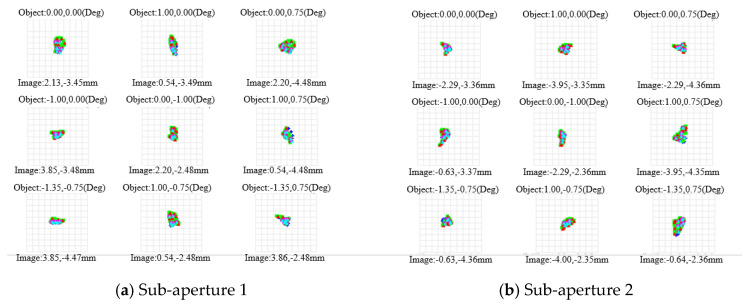

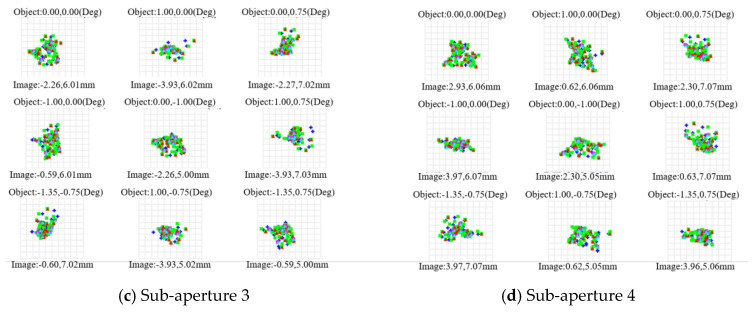
Spot diagram of aperture-divided off-axis simultaneous polarization super-resolution imaging optical system. (**a**) Sub-aperture 1; (**b**) Sub-aperture 2; (**c**) Sub-aperture 3; (**d**) Sub-aperture 4.

**Figure 12:**
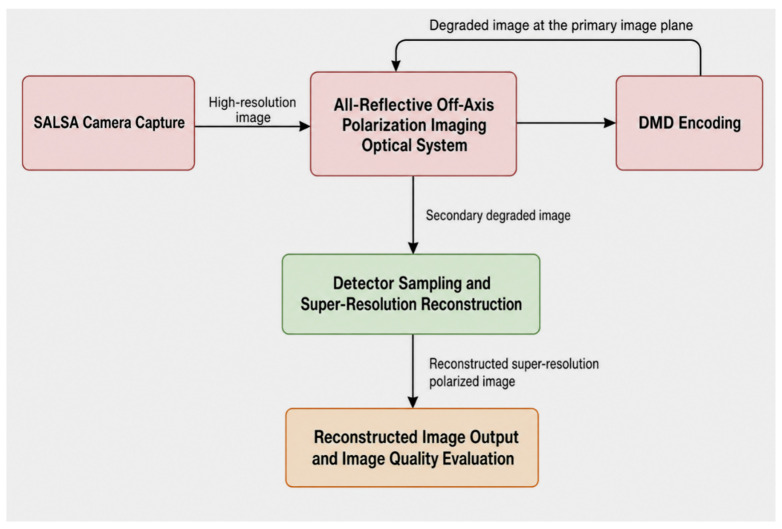
Simulation workflow.

**Figure 13:**
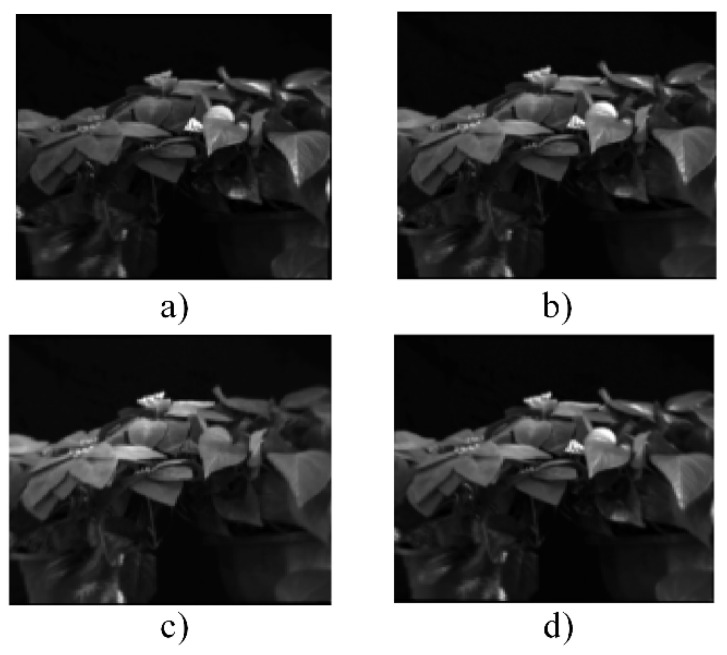
Low-resolution image.(**a**) 0°, (**b**) 45°, (**c**) 90°, (**d**) 135°.

The design performance of the telescope objective, relay reflection system, and overall optical system is evaluated using several metrics, including MTF curves, defocus spot radius, ray aberration curves, and ray trace distributions. The evaluation results are presented in Figure 7, Figure 8, Figure 9, Figure 10, Figure 11, Figure 12 and Figure 13. Figure 7 illustrates the MTF curves for each sub-aperture of the telescope objective.

Figure 8 shows the optical path diagram, MTF curve, spot diagram, and grid distortion for the relay reflection system. These results demonstrate that the aberrations of both subsystems are effectively corrected, ensuring the reliability of subsequent compressed sensing image reconstruction.

Figure 9 presents the overall MTF of the optical system, where the MTF values for each sub-aperture at the system cutoff frequency of 20 lp/mm exceed 0.4.

Figure 10 displays the ray trace diagram at the DMD and the infrared polarization focal plane detector, where the four sub-apertures are positioned optimally in each field of view. The maximum deviation of rays from the ideal focus at the center of each field of view is less than one pixel, ensuring accurate super-resolution coding and reconstruction performance. Figure 11 shows the spot diagram for each sub-aperture, with the root mean square (RMS) radius of each optical spot smaller than the detector pixel size.

### 4.2. Tolerance Analysis

The optical system tolerances are classified into assembly and manufacturing tolerances. The assembly tolerances of the reflective system include translation tolerances along the *x*, *y*, and *z* axes, as well as tilt tolerances about these axes. The manufacturing tolerances include curvature radius tolerance, M_2_ surface coefficient tolerance, and freeform surface shape tolerance. The distribution of these tolerances is presented in Table 4. Current manufacturing capabilities are sufficient to meet the required processing and assembly precision for this system.

The system is evaluated using the RMS wavefront aberration as the final criterion. A Monte Carlo tolerance analysis is performed with 500 iterations, and the tolerance parameters are distributed as shown in Table 5. According to the Monte Carlo analysis, 98% of the samples have an RMS wavefront aberration of less than 0.08λ (λ = 8 μm), which meets the imaging clarity requirements [20,21]. When performing multi-channel image fusion, in order to ensure that the image is not missing, it is necessary to ensure that the center coaxiality deviation between the two channels is less than one pixel size. By using a pixel size and the effective focal length of the optical system, it can be calculated that the coaxial deviation angle of the system should be less than 0.015 °.

## 5. Simulation Experiments

The reconstruction algorithm in compressed sensing is essentially an optimization problem; more specifically, it involves solving an $l_{1}$-norm minimization problem. The $l_{1}$ norm is used to approximate the $l_{0\;}$ norm. In this paper, an improved block OMP algorithm is adopted for reconstruction. Based on the off-axis sub-aperture polarization super-resolution imaging optical system, super-resolution reconstruction simulations based on compressed sensing were carried out, and the simulation workflow is shown in Figure 12.

The simulation workflow is as follows: first, high-resolution polarization scene images are imported into the Zemax database. Since high-resolution infrared polarization images are difficult to obtain and the main evaluation target is resolution enhancement, four visible-light polarization images of the same scene, captured by a SALSA polarization camera at 0°, 45°, 90°, and 135°, are used as inputs. Next, Zemax image simulation is used to generate the primary degraded image at the first image plane after the target passes through the telescope objective. Then, block-based sampling and encoding are performed at the DMD according to device resolution (1536 × 1152 micromirrors; 768 × 576 per sub-aperture; detector resolution 192 × 144, i.e., 4 micromirrors correspond to 1 detector pixel). In MATLAB 2020, the compressed encoding process is simulated by applying block-wise measurement matrices to the primary degraded image; during reconstruction, each block is restored separately, stitched together, and corrected for edge effects (the measurement matrix is expanded from multiple 4 × 4 random Gaussian matrices). The encoded image then passes through the relay reflective system, undergoing secondary degradation and downsampling; optical simulation yields degraded images of 192 × 144 for each sub-aperture. Steps of encoding and secondary degradation are repeated for all four sub-apertures to obtain multiple encoded, downsampled detector images. Finally, taking sub-aperture 1 as an example, OMP reconstructs the 192 × 144 image to 768 × 576, achieving 4× super-resolution, and the final image quality is evaluated using PSNR and SSIM.

Because this optical system has multiple apertures, Zemax’s DDE function is used to reduce workload by linking MATLAB code with a running Zemax session and reading data directly from Zemax. For full-system simulation, the Zemax file of the designed all-reflective broadband polarization imaging optical system is opened in advance for MATLAB calls. Through this Zemax–MATLAB linkage, four original polarization scene images at 0°, 45°, 90°, and 135°are imported. After degradation through the optical system, four downsampled low-resolution images are obtained, and super-resolution reconstruction is then performed on these low-resolution images. Finally, four reconstructed images with resolutions four times higher than that of the detector are obtained, and the reconstruction performance is evaluated using SSIM and PSNR, as shown in Table 6.

The low-resolution image is shown in Figure 13.

**Figure 14:**
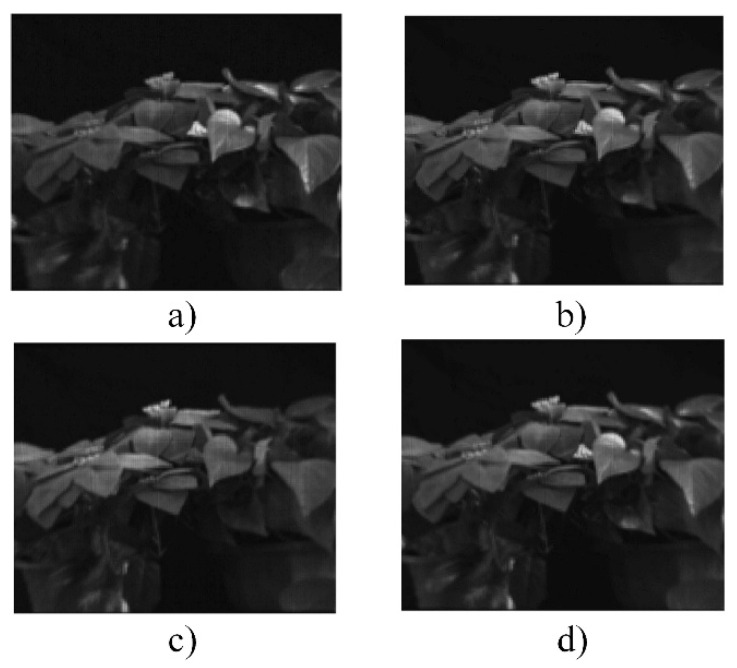
Reconstruction results (**a**) 0°, (**b**) 45°, (**c**) 90°, (**d**) 135°.

The reconstruction results are shown in Figure 14.

According to Equation (1), the reconstructed polarization images at 0°, 45°, 90°, and 135° were further combined to obtain the super-resolved Stokes images S0, S1, and S2, where S3 was set to zero for linear polarization-only measurement.

**Figure 15:**
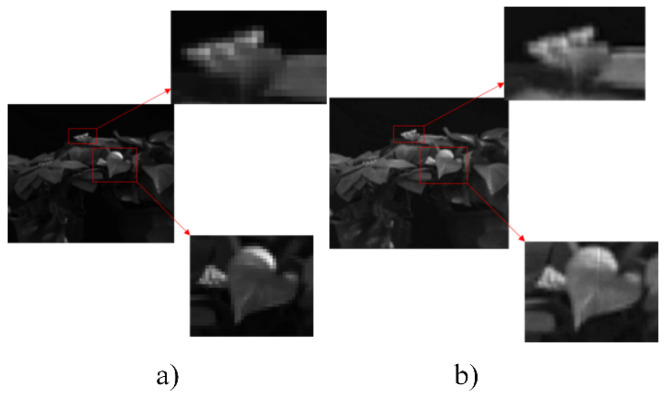
Detail Comparison Between Low-Resolution and Reconstructed Images. (**a**) Magnified detail of the low-resolution image. (**b**) Magnified detail of the reconstructed image.

A comparison between the low-resolution images and the reconstructed high-resolution images shows that the low-resolution images suffer from a pronounced mosaic effect, as shown in Figure 15. For example, in the low-resolution images, the flower core is almost indistinguishable, whereas in the reconstructed images, clear structural layers between the stamens can be identified. In the large-leaf regions, the leaf edges in the low-resolution images are severely blurred, while the reconstructed images show significantly improved edge detail and overall fineness. These results verify the effectiveness of the compressed-sensing-based super-resolution reconstruction method proposed in this paper and demonstrate a clear resolution enhancement for low-resolution images.

## 6. Conclusions

This paper proposes an aperture-divided off-axis polarization-sensitive super-resolution imaging system, realized using an aperture-divided reflective freeform optical system and a DMD encoder. The system offers several advantages, including applicability across various optical wavelengths, simultaneous imaging of multiple polarization states, single-detector operation, high resolution, and ease of miniaturization. Additionally, the design principles and methods for an aperture-divided off-axis reflective freeform optical system incorporating a DMD have been studied and developed. The classical W-W theory in Optical Principles is further developed to derive a reflective W-W differential equation suitable for reflective systems, capable of eliminating various aberrations. Additionally, by integrating Seidel’s aberration theory, the solution to the W-W equation is iteratively adjusted to satisfy the boundary conditions for distortion elimination, resulting in an optical initial structure that simultaneously corrects spherical aberration, coma, astigmatism, and distortion. A merit function for image quality is established to strictly control the position of the chief ray’s intersection points at the intermediate and final image planes for each sub-aperture across all fields of view. This effectively suppresses the mismatch errors during the super-resolution reconstruction process at the optical level. The design of an aperture-divided off-axis reflective super-resolution imaging optical system with four sub-apertures has been completed. Each mirror surface is designed as an X-Y polynomial freeform surface. The system features a large relative aperture (F# = 2.5) and a compact structure. At both the intermediate image plane (DMD) and the final image plane, the image quality for each sub-aperture and field of view is near the diffraction limit, meeting the imaging quality requirements for each polarization channel. The design principles and methods proposed fill the gap in the theoretical framework for wide-band simultaneous polarization super-resolution imaging optical systems. This approach addresses the issues of low design efficiency and poor reliability commonly encountered when applying traditional design methods to such specialized systems. For the practical manufacturing of this specialized optical system, tolerance analysis is a crucial step. Therefore, the next step is to develop a tolerance model for the system, taking into account the current capabilities in freeform surface fabrication. This will involve allocating tolerance values for the curvature radius, polynomial coefficients, mirror spacing, eccentricity, and tilt about the x-axis, in preparation for the realization of the actual system. Because the DMD encodes each polarization channel sequentially, improved spatial super-resolution is achieved at the cost of reduced temporal resolution. In addition, maintaining optomechanical alignment and thermal stability over the broad 3–14 μm MWIR–LWIR band remains an important practical challenge for system implementation. It should be noted that the present work focuses on optical design, aberration correction, and simulation-based feasibility evaluation. Prototype fabrication, real-scene imaging experiments, experimental polarization calibration, reconstruction validation using measured data, and real polarimetric tests are beyond the scope of the present study and will be carried out in future work.

## Figures and Tables

**Table 1:** Comparison with prior aperture-divided polarimeters.

Reference	Spectral Band	F/#	EFL/FOV
Pezzaniti & Chenault [3]	MWIR	-	-
Moultrie et al. [4]	VIS	-	-
Leon et al. [5]	632.8 nm	-	-
Huang [8]	MWIR	F/2	f = 68 mm, 4° × 3.2°
Wang et al. [9]	3.7–4.8 μm	F/4	f = 200 mm, 2° half-FOV
Liu et al. [10]	240–390 nm	-	-
This work	3–14 μm	F/2.5	EFL 100 mm, 2.7° × 2.0°

**Table 2:** Specification of aperture-divided off-axis simultaneous polarization super-resolution imaging optical system.

Parameter	Specification
Effective focal length	100 mm
Entrance pupil diameter	40 mm
Field of view	2.70° × 2.00°
F number	2.5
Wavelength	3–14 μm
MTF	>0.4@20 lp/mm
Pixel number and size of detector	384 × 288; 25 μm
Pixel number and size of DMD	1536 × 1152; 10.8 μm

**Table 3:** Profile data points of M_1_ and M_2_.

N	z1	y1	z2	y2
1	−0.008126	2.5	0.046	3.549
2	−0.018	3.75	0.104	5.324
3	−0.026	4.499	0.15	6.389
4	−0.073	7.497	0.415	10.647
5	−0.129	9.994	0.739	14.195

**Table 4:** Mirror parameters.

Surface	Surface Type	Radius/mm	Thickness/mm
Objective	Free-form surface	−301.483	−150
M_1_	Free-form surface	−439.79	150
M_2_	Free-form surface	180	−144.122

**Table 5:** Tolerance distribution of optical system.

Tolerance Type	Tolerance Name	Telescope Objective	M_2_	M_3_
Assembly Adjustment Tolerance	X Displacement/mm	-	0.08	0.1
	X Tilt/(‘)	-	1/3	1/2
	Y Displacement/mm	-	0.08	0.08
	Y Tilt/(‘)	-	1/3	1/3
	Z Displacement/mm	-	0.2	0.2
	Z Tilt/(‘)	-	1/4	2/3
Machining tolerance	Radius of curvature/mm	0.2	0.3	0.3
	Quadratic surface coefficients	0.1%	0.07%	0.2%
	Surface error	λ/50	λ/50	λ/50
	Maximum coaxial deviation angle/(°)	0.015	0.015	0.015

**Table 6:** Reconstruction evaluation metrics.

	SSIM	PSNR (*DB*)
0°	0.55251	30.8742
45°	0.64749	38.3686
90°	0.63449	43.5578
135°	0.57775	36.6583

## Data Availability

The data presented in this study are available in article. Further inquiries can be directed to the corresponding author.
